# Research hotspots and frontiers of profile technology applied in education—a visualization analysis based on CiteSpace

**DOI:** 10.3389/fpsyg.2025.1668749

**Published:** 2026-01-16

**Authors:** Lijing Zhang, Yu Wang, Jia Li, Jianzhan Wang

**Affiliations:** 1 Marxism School, China Pharmaceutical University, Nanjing, China; 2 Party Committee Office, China Pharmaceutical University, Nanjing, China; 3 School of Foreign Studies, Nanjing University of Posts and Telecommunications, Nanjing, China

**Keywords:** CiteSpace, digital education, learner profile, personalized learning, profile technology

## Abstract

This study takes 300 literatures (from 2014 to 2024) included in the Web of Science Core Collection (mainly SSCI and SCI-E) as the data source and uses CiteSpace 6.3.R1 software to analyze the evolution of Profile Technology Applied in Education (PTAE). The research identified three core clusters: learner analysis and recommendation, intelligent technology-driven Educational Data Mining (EDM), and governance of the blended learning ecosystem. It reveals the deep transformation path of the field from basic feature characterization through data-driven prediction to a panoramic intelligent ecosystem. The research finds that the core research model is transitioning from static portrayal to a dynamic and precise intervention mechanism based on a panoramic learning analytics framework, establishing the underlying logic of modern adaptive systems Based on the findings, this paper explores algorithmic fairness and ethics challenges, and proposes practical strategies for building an ecologically adaptive intelligent educational environment.

## 1 Introduction

The concept of profiling originated from the term “User Persona,” proposed by Alan Cooper, the father of interaction design. It refers to a target user model constructed based on real data, representing the characteristics of real users. This concept was initially applied in the business field, where data mining technology was used to analyze user backgrounds and behavioral patterns to improve product design and tap into the value of data (Aoyama, 2005). Researchers have transferred profiling to education, giving rise to core concepts such as learner profile, student portrait, and teacher portrait. Among them, User Persona is its technical prototype and theoretical basis; Learner Profile, a general term for its application in the field of education, denotes the process of digitally representing learners’ cognitive characteristics, behavioral patterns, emotional states, and social environments through multi-dimensional data collection and analysis (Ni et al., 2024). Student Portrait is a data analysis technology that can quickly and accurately analyze the characteristics of students. It aims to process and analyze various types of student data for certain purposes to form a student portrait model, which can be regarded as the refinement and practical application of learner profile in specific student groups (Hu and Chen, 2020; Dinh and Harada, 2013). Teacher Portrait derives from the concept of User Profile, but few studies explicitly put forward this concept. Previous research tended to interpret teacher profiling as a data-based framework description. The current description is not comprehensive, as it only focusing on teachers’ effectiveness or their interests (Hu et al., 2024). Educational Portrait, a general term for the application of Profile Technology in educational scenarios, defines the technology and process of constructing digital characteristic models to support educational decision-making and personalized services—achieved through the collection and analysis of multi-dimensional data (e.g., academic, life, and behavioral data) around key educational subjects such as students and teachers (Zhai et al., 2021). With Digital Education transformation driven by artificial intelligence, Profile Technology has evolved into a key link connecting intelligent technology and educational practice. Centered on the theory of personalized learning as its core pedagogical foundation, it relies on methodological support of learning analytics and educational data mining, thereby emerging as a critical technical underpinning for achieving the precise representation of learning characteristics and propelling the scientization of educational decision-making. Previously, the research of scholars from all over the world on PTAE mainly centered on the conceptual definition, model construction, personalized learning, and course recommendation. While the limitation lies in the fact that most of them are based on their own country’s conditions and confined to a single disciplinary area, with few scholars conducting macroscopic, systematic, and quantitative analyses of the overall research hotspots and trends through bibliometric software. Thus, creating a clear research gap that this research aims to fill.

To address the limitations of the aforementioned studies and systematically grasp the frontier dynamics of this field, this paper, based on the literature on PTAE included in the Web of Science (WOS) database during the period of 2014-2024, optimally adopts the bibliometric method and uses software CiteSpace (6.3.R1) to conduct visual analysis. This research conducts quantitative analyses of annual publication output, publication trends, author and institutional distributions, keyword co-occurrence and clustering, keyword timelines, and burst keywords to construct a literature map. It seeks to systematically organize research achievements related to portrait technology empowering education, explore its evolutionary process, grasp its research hotspots, and predict its future development trends, thereby providing references for theoretical research and practical applications in this field.

## 2 Research methodology and data sources

In this study, the sub-databases of the American comprehensive online literature database Web of Science (WOS), namely, the Social Science Citation Index (SSCI) and Science Citation Index Expanded (SCI-Expanded), were searched in November 2025. CiteSpace (6.3.R1) software was used for quantitative analysis. The types of literature selected include research articles, review articles and conference articles, with the time range set from January 2014 to December 2024, and the language chosen as “English.” The search query was: TS = ((“learner portrait” OR “learner profile” OR “student profile” OR “teacher portrait” OR “learner persona” OR “educational portrait”) AND “(education” OR “e-learning” OR “online learning” OR “MOOCs”))

Due to the weak relevance of some literature, the research field was limited to education research, computer science and other fields with strong relevance. After manual reading and review of the title, abstract and key words of the literatures, a total of 300 literatures were obtained. This study analyzes 10 years span from 2014 to 2024, with the core being the ability to systematically present the changes in research hotspots and achieve dual-dimensional adaptation of policies and technologies. In terms of policy, this span completely covers the entire cycle from the planning and formulation to the implementation of the global digital transformation of education. Initiated by the launch of the European Union’s “Erasmus+” program and Germany’s *Digital Agenda* in 2014, which kickstarted educational digitalization, subsequent AI education strategies were introduced by countries such as China, the United States, and the United Kingdom. The popularization of electronic textbooks in Japan in 2024 marked the implementation of this practice. From a technological perspective, this timeframe accurately aligns with three phases of Profile Technology’s usage in education: basic application, rapid development, and in-depth adaptation. It not only encompasses the deep integration of AI, data mining, and education but also captures the driving role of emerging hotspots such as emotion analysis and generative AI in shaping research themes, thereby providing a stable reference for interdisciplinary comparisons.

**FIGURE 1:**
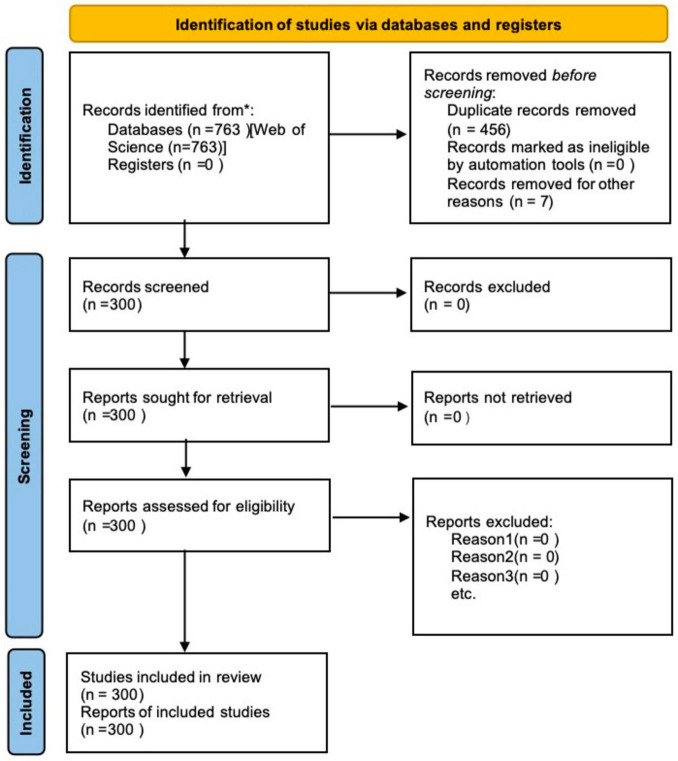
Literature retrieval strategy and screening flowchart based on the PRISMA 2020 statement.

The specific process of literature retrieval and screening follows Preferred Reporting Items for Systematic reviews and Meta-Analyses (PRISMA) standard. Detailed steps and the number of literature at each stage are shown in Figure 1.

## 3 Research hot spots and frontier analysis

### 3.1 Publication year and volume analysis

**FIGURE 2:**
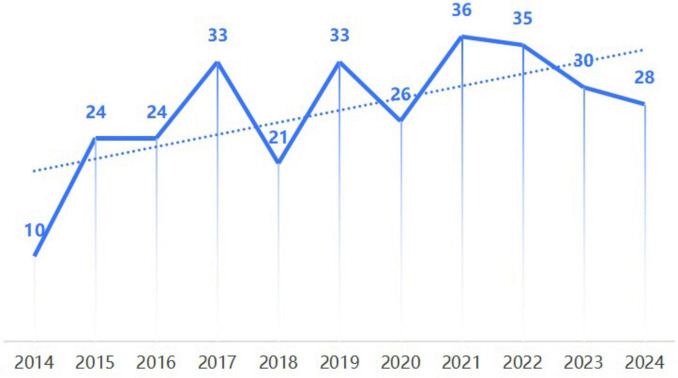
Annual publication volume and trend chart of profile technology in education research from 2014 to 2024.

The number of publications is an effective indicator that reflects the research heat of a specific topic over a certain period of time, and it can intuitively show the development trend of the research topic. Based on the literature downloaded from the WOS database, a distribution chart of the years of relevant research plotted (Figure 2).

The analysis of publication volume (Figure 2) reveals that the annual number of international publications on Profile Technology has fluctuated but generally shown an upward trend during the decade from 2014 to 2024. Among them, a peak of 36 articles was reached in 2021. The emergence of this peak is the result of the combined effect of global policy coordination, demand traction and technological empowerment. The G7 Group, which accounts for 43% of the world’s GDP (Statista, 2022), has taken the digital economy as its core strategy. At its 2021 summit, it proposed building an exclusive digital ecosystem (The White House, 2021); the Biden administration, in conjunction with member states, unveiled the Build Back Better World (B3W) initiative, expanding the application scenarios of Profile Technology through cooperation in global digital infrastructure construction (G7 Research Group, 2021). Chinese policies have further reinforced this trend-14th Five-Year Plan for Digital Economy Development issued in 2021 laid out requirements for digital supply in the field of education (The State Council, 2021), and the Ministry of Education listed educational digitalization as a key task in 2022 (Ministry of Education, 2022). The alignment of this deployment with global strategic trends has opened up abundant empirical application space for Profile Technology and promoted the international academic community to converge on this theme.

Statistics show that among the literature, 239 are research papers, 58 are conference papers, and 3 are review papers, and the researchers mainly come from countries such as China, Morocco, Spain, the United States, India, Tunisia, Australia, and other countries. In terms of disciplines and majors, researchers mainly focus on computer science education, followed by psychology education, psychiatry, neuroscience, neurology and economics. Researchers from countries like the United States, Morocco, and the United Kingdom emphasize electronic learning recommendation systems (Bourkoukou et al., 2017; Colchester et al., 2017); Tunisian and French researchers make new explorations in the application of gamified learning (Missaoui and Maalel, 2021; Lavoué et al., 2018); while Chinese researchers mainly focus on topics such as personalized learning (Peng et al., 2019), learner motivation influences (Moore and Wang, 2021), data mining clustering (Kausar et al., 2018), learner modeling (Li, 2018; Liang et al., 2023), emotional experience of online learners (Zhao and Song, 2022), and deep learning (Xu et al., 2022). These research directions enhance the personalization and intelligence of education through a data-driven approach, promote the fairness and democratization of education, and thus lift up the overall quality and level of education.

### 3.2 Analysis of research authors and main institutions

#### 3.2.1 Analysis of co-authors

**FIGURE 3:**
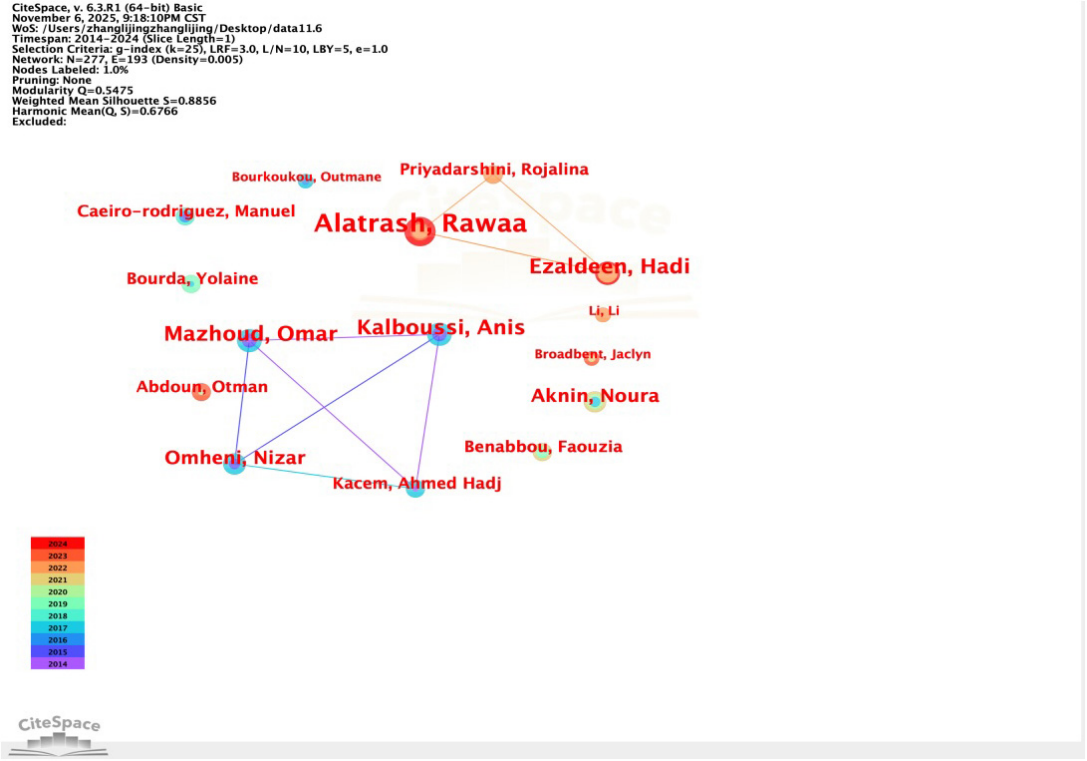
Knowledge graph of core author collaboration network in PTAE research from 2014 to 2024.

In the CiteSpace software, set node type as author, the time slice as 1 year, g-index as the node screening criterion, scale factor k as 25, and choose None as the Pruning method, the knowledge map of the main study authors can be obtained (Figure 3). The results show that 277 author nodes (*N* = 277), the amount of connectivity between authors is 193 (*E* = 193), and the network co-occurrence density is 0.005 (*D* = 0.005) were generated in this graph, which reflects the fact that between 2014 and 2024, although there are many authors internationally conducting research on PTAE and some scholars have collaborative relationships with each other, the concentration of cooperation is relatively low. Among them, the authors who have published more articles and have significant cooperative relationships are Alatrash Rawaa, Mazhoud Omar, Kalboussi Anis, Ezaldeen Hadi, Li Li, Kacem, Ahmed Hadj, Broadbent, and Jaclyn. These authors are from the University of Duisburg-Essen, Universite de Kairouan, CV Raman Global Univ, Shanghai University of Political Science and Law, Sfax University, Deakin University, and Kairouan University, and their main focus is on learner modeling, personalization, and they primarily focus on topics like learner modeling, personalized modeling, and educational recommendation systems. In these collaborative relationships, there are few scholars from China, indicating that it is necessary and urgent to strengthen international cooperation in this research area.

#### 3.2.2 Publications and citations

Analyzing the number of publications and research directions of core authors helps better capture the overall trend of the discipline. In this paper, the Price formula is employed to select candidates for core authors (Price, 1970). The formula is:


$$M_{p}=0.749*\sqrt{N_{p\,m\,a\,x}}$$

M_p_ refers to the lowest number of articles published by core author candidates, N_pmax_ refers to the highest number of articles published by authors within the selected literature (Liu et al., 2015). The result shows that *M_p_* is 2.11, taking an integer of 2, which reveals that the core author candidates have no less than 2 publications in the period 2014-2024. Citation is also an important indicator of the quality of the paper, as its frequency indirectly indicates the impact and attention of the paper (Li, 2008). In this paper, the minimum number of publications is calculated according to Price’s formula (Price, 1970):


$$M_{c}=0.749*\sqrt{N_{p\,m\,a\,x}}$$

*M_c_* refers to the minimum number of citations for a single paper, *N*_*pmax*_ refers to the maximum number of citations for a single paper.

According to statistics, the maximum number of citations is 395, Mc≈14.9, rounded to 15, and the core authors who satisfy the above two indexes are Broadbent Jaclyn (citation frequency *395), Alatrash Rawaa (citation frequency *37), Ezaldeen Hadi (citation frequency *37). These authors are mainly from Deakin University, University of Duisburg-Essen and CV Raman Global University. To further quantitatively present the academic output characteristics and representative achievements of the core author groups mentioned from a macro perspective, this research has collated the data of high-yield authors and highly cited literature in this field (Tables 1, 2). Table 1 reveals the research focus and academic influence of the core authors, and Table 2 sorts out the key literature that laid the theoretical foundation of this field.

**TABLE 1:** Core authors’ academic output, influence, and research directions in this field.

Ranking	Author name	Affiliation	Amount of published papers	Total citation frequency	Core research directions
1	Alatrash Rawaa	University of Duisburg-Essen	8	108	Learner modeling knowledge graph
2	Broadbent Jaclyn	Deakin University	4	518	Self-regulated learning
3	Kalboussi Anis	Universite de Kairouan Universite de Sfax	5	50	Remote learning Recommendation System Learner modeling
4	Ezaldeen Hadi	CV Raman Global Univ	5	120	Deep learning Blended learning Personalized learning

**TABLE 2:** High-impact representative literature.

Ranking	Authors and years	Titles of paper
1	Broadbent, 2017	Comparing online and blended learner’s self-regulated learning strategies and academic performance
2	Ezaldeen et al., 2022	A hybrid E-learning recommendation integrating adaptive profiling and sentiment analysis

Alatrash Rawaa has the largest number of publications and has a total high citation frequency of 108. Most of Alatrash’s works are related to topics such as learner modeling, optimization of e-learning recommendation systems, and emotional analysis of online learners. In his article published in 2022, he proposed the Enhanced e-Learning Hybrid Recommender System (ELHRS) that aims to recommend appropriate learning content based on the personal needs of learners (Ezaldeen et al., 2022). More specifically, this system developed a new model for automatically inferring learner configuration profile semantics, which links e-learning materials and terminology together by analyzing learner behavior and semantic relationships, and adaptively associating learning patterns and rules. His study also innovatively utilized DBpedia and WordNet for semantic-based terminology expansion, along with a sentiment analysis model to predict the ratings of e-learning resources, as well as the hybrid language model developed by the qualitative Natural Language Processing (NLP) approach and the customized Convolutional Neural Network (CNN) markedly improved the accuracy of lexical expressions up to 89.1%. The recommender system not only considers learners’ personal preferences, but also emphasizes the key role of sentiment analysis in personalized and intelligent learning recommender systems to help learners acquire desired content at the right time, thus improving the efficiency of resource utilization, which lays a good foundation for subsequent research on PTAE.

According to Price Law, when the number of articles published by core authors reaches 50% of the total number of articles, it means that a core group of authors has been formed in the field (Liu et al., 2015). However, the above core authors only account for 10% of the total number of publications, which is notably lower than this standard, so it can be seen that the current research on this field has not yet constituted a core group of authors, and the cooperation is relatively decentralized. Meanwhile, this also indicates that the international authors focusing on this field are obviously diversified, but there is still a lack of effective cooperation.

#### 3.2.3 Research institutions

As can be seen from the top fourteen institutions in terms of the number of publications on PTAE theme (Table 3), Abdelmalek Essaadi University of Tetouan currently own the highest number of publications on this research topic abroad, with 22 publications in each of them during the period 2014-2024. Mohammed V University in Rabat and Hassan II University of Casablanca, which ranked second and third, published 16 and 10 papers, respectively. The authors are mainly concentrated in universities, mainly from China, Morocco, the United States, India, Australia, Spain, the United Kingdom, Tunisia, France and other countries. Among them, China has the highest number of publications, with 60 papers, followed by Morocco (56), Spain (30), the United States (27), with Australia having relatively fewer (14) (Table 4). The main focal points of these countries include course recommendation, self-regulation, learner model construction, the role of emotional experience and sentiment analysis, deep learning, personalized learning, data mining methods, etc. The main research institutions from China include the Education University of Hong Kong (EdUHK), whose main focuses concentrate on personalized learning, deep learning, MOOCs research, online learners’ emotional experience, the impact of learner motivation, the democratization of higher education, etc.

**TABLE 3:** Institutions with a publication volume of 4 or more articles and their publication time from 2014 to 2024.

Serial number	Publication count	Time	Research institution
1	22	2014	Abdelmalek Essaadi University of Tetouan
2	16	2014	Mohammed V. University in Rabat
3	10	2017	Hassan II University of Casablanca
4	10	2014	Universite de Sfax
5	6	2017	Ibn Tofail University of Kenitra
6	5	2019	East China Normal University
7	5	2014	Universite de Kairouan
8	4	2017	Education University of Hong Kong (EdUHK)
9	4	2016	Ecole Nationale dingenieurs de Sfax
10	4	2015	Pontificia Universidad Catolica de Chile
11	4	2022	CV. Raman Global Univ
12	4	2015	Jeju National University

**TABLE 4:** National distribution and publication volume from 2014 to 2024 (unit: part).

Serial number	Country	Publication count
1	Peoples R China	60
2	Morocco	56
3	Spain	30
4	USA	27
5	India	25
6	Tunisia	20
7	France	15
8	England	15
9	Australia	14

### 3.3 Analysis of research hotspots

#### 3.3.1 Keyword co-occurrence analysis

**FIGURE 4:**
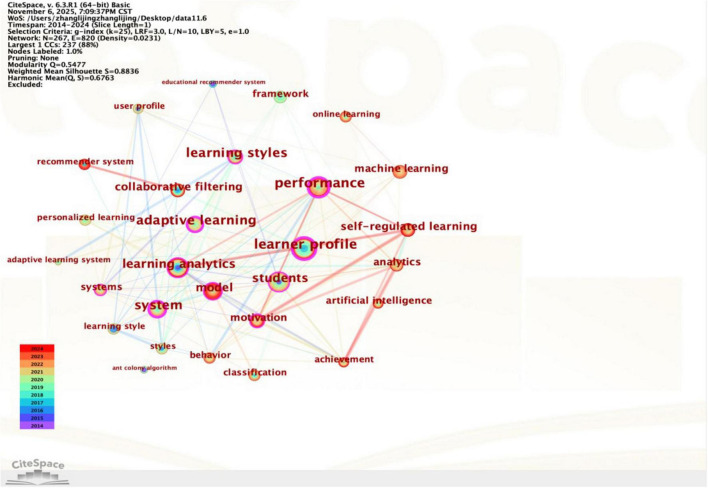
Keyword co-occurrence network and core node association graph.

Keywords are a highly condensed and summarized representation of the research topic, helping readers quickly grasp the core content of the study, and have positive significance for analyzing research hotspots and predicting development trends. The betweenness centrality calculated by CiteSpace represents the number of times a node acts as a bridge on the shortest path between two other nodes, In the context of bibliometrics, nodes exhibiting high betweenness centrality (typically > 0.1) often signify pivotal turning points or transformative concepts that bridge distinct research clusters. In-depth analysis and interpretation of these key nodes can predict research hotspots in the field (Ren, 2021). Importing 300 English literature articles into CiteSpace, setting the node type to keywords, and the time slice to 1 year, while keeping the rest of the settings as default, the software generated a keyword co-occurrence network knowledge graph containing 267 nodes (*N* = 267), 820 links (*E* = 820), and a density of 0.0231 (*D* = 0.0231) (Figure 4).

According to the Donohue equation (Donohue, 1973):


$$T=\frac{\left[-1+\sqrt{\left(1+8\,I\right)}\right]}{2}$$

“I” refers to the number of keywords and “T” refers to the threshold of high-frequency keywords (Wei and Tang, 2016). High-Frequency words in this research field can be quickly found by calculating the threshold value. According to CiteSpace, the value of I is 267, and T is calculated to be approximately 22.61, which results in a list of high-frequency keywords with a frequency greater than 22 (Table 5). Therefore, combining the keyword co-occurrence network knowledge graph (Figure 4) and the high-frequency keyword list (Table 5), the keyword “learner profile” can be obtained by filtering the nodes with a frequency of occurrence greater than 22 and a node centrality greater than 0.1. Other high co-occurrence frequency keywords such as “performance,” “adaptive learning,” “system,” “learning analytics,” “model” etc., do not have frequency higher than 22, but they also reflect the main concerns of the research field to a certain extent.

**TABLE 5:** High-frequency keywords (frequency ≥ 22) along with their betweenness centrality and first appearance year related to PTAE research.

Serial number	Frequency	Betweenness centrality	Occurrence year	Keyword
1	22	0.22	2016	Learner profile
2	21	0.28	2016	Performance
3	19	0.22	2014	Adaptive learning
4	17	0.23	2016	System
5	15	0.10	2015	Learning analytics
6	13	0.20	2017	Model

Scholars from all over the world are conducting creative research and innovative exploration on PTAE from different perspectives based on local realities. The analysis results show that “learner profile” has the highest frequency of occurrence, which appears in 22 papers and accounting for 7.3% of the total volume, with the highest number of publications both in 2017 and 2019, each with six articles; the keyword with the greatest betweenness centrality is “performance,” which appears in 17 papers and accounting for 5.6% of the total volume, the year with the highest number of publications using this keyword is 2022, with a total of 5 papers.

Such a high-frequency result is highly consistent with the learner-centered new educational paradigm advocated by international instructional design expert Professor Charles M. Reigeluth. Focused on learners’ individuality and learning processes, this paradigm advocates a shift from standardized assessment to the evaluation of knowledge and skill mastery, and from summative assessment to formative assessment (Liu et al., 2017). Learner Profile, as a key technical underpinning for realizing personalized teaching and process-oriented tracking through the integration of multi-dimensional data, serves as the core carrier of the technology-empowered educational innovation model. It not only aligns with the educational principles based on goal achievement and task orientation but also provides a data-driven practical pathway for the implementation of individualized learning—thus explaining its sustained attention in global research.

At the practical level, scholars, centering on this core concept, have achieved in-depth extension of Profile Technology from theoretical models to application scenarios through multi-dimensional technical means. In order to tackle the challenges of information overload and resource mismatch, Madani, Youness, Bourkoukou Outmane and other scholars combined social filtering, collaborative filtering and reinforcement learning methodology to construct a recommendation model capable of automatically selecting optimal resources according to learners’ knowledge, skills, and preferences. This model has effectively improved the learning adaptability and path planning quality in online and remote environments (Madani et al., 2020; Bourkoukou and El Bachari, 2016). Meanwhile data insights are widely used to drive teaching interventions. Badal Yudish Teshal utilized machine learning algorithms such as random forests to analyze interaction data, achieving accurate prediction of academic performance and participation (Badal and Sungkur, 2023). Liang introduced the Jaccard coefficient and an intelligent guidance model to conduct in-depth cleaning and modeling of learning attitudes and behavioral characteristics, providing elaborate profiling support for improving e-learning experience (Liang et al., 2017). Furthermore, addressing learners’ dynamic needs and independent development, Premlatha, K. R, Almohammadi, Khalid, and other scholars created an adaptive system designed to track navigation logs and emotional states to dynamically update profiles (Premlatha et al., 2016; Colchester et al., 2017). Winne (2021), from the perspective of self-reflection, proposed the integration of process tracking data to generate transparent feedback, thereby helping learners optimize their self-regulation strategies.

#### 3.3.2 Keyword cluster analysis

**FIGURE 5:**
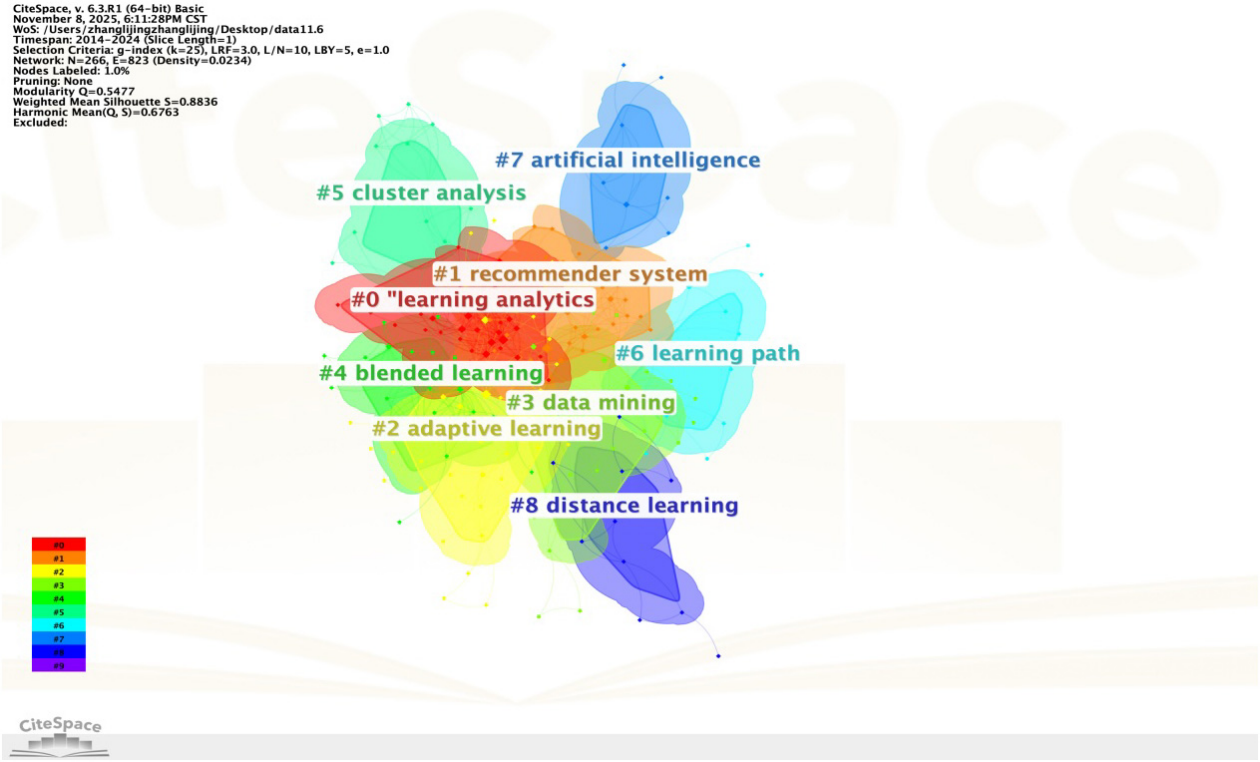
Keyword cluster distribution and the attribution map of three major research fields.

Based on co-occurrence network analysis, keyword cluster analysis uses the LLR algorithm to group closely related high-frequency keywords into one category. It provides a high overview of the research hotspots in a certain field, macroscopically presents the overall research, grasps the current research direction, and offers a basis for further detailed analysis (Yan et al., 2022). This paper clusters WOS literature to obtain the keyword cluster graph. The cluster modularity value is a credibility metric for evaluating keyword clustering knowledge graph (Figure 5). The mean Silhouette (S) value quantifies the homogeneity of the cluster members, where a value approaching 1 signifies high intra-cluster consistency. The results of the cluster analysis show that the module value of Q is 0.5477, and the value greater than 0.3 indicates that the cluster structure is significant, and the average silhouette S is 0.6763, which is greater than 0.5, implying that the results of this clustering are reasonable.

The keyword clustering graph displays a total of 9 clustering categories, which are “#0 learning analytics, #1 recommender system, #2 adaptive learning, #3 data mining, #4 blended learning, #5 cluster analysis, #6 learning path, #7 artificial intelligence, #8 distance learning.” The original clustering results can be manually organized and summarized into three major categories combined with clustering labels and other core keywords in the same category, thereby creating a keyword clustering analysis graph (Table 6).

**TABLE 6:** Keyword clustering categories and corresponding research topics.

Cluster topic	Cluster number	Cluster name
Learner analysis and personalized recommendation	#0, #1, #2, #6	#0 learning analytics #1 recommender system #2 adaptive learning #6 learning path
Intelligent technology	#3, #5, #7	#3 data mining #5 cluster analysis #7 artificial intelligence
Diverse learning scenarios	#4, #8	#4 blended learning #8 distance learning

The first category is learner analytics and personalized recommendation research, which mainly includes #0 learner analytics, #1 recommender #2 adaptive learning, #6 learning path and the other four clusters, involving high-frequency keywords like “MOOCs,” “attitudes,” “learn style,” “course resources,” etc. Existing research mainly focuses on dealing with the issues of information overload and the lack of personalized support in online learning environments. After an in-depth review of the literature, current research can be categorized into three core academic domains: intelligent recommendation systems and dynamic adaptive modeling, computational feature analysis and self-regulated learning (SRL) modeling, and learning analytics-driven resource adaptation, feature profiling, and precision intervention.

##### 3.3.2.1 Intelligent recommendation systems and dynamic adaptive modeling

Research in this field focuses on leveraging computational intelligence technologies such as deep learning and reinforcement learning to construct multi-scale dynamic learner profiles, aiming to overcome the exploration-exploitation dilemma in recommendation systems. To tackle the insufficient dynamic adaptability of recommendation strategies in online learning, Lin et al. proposed Hierarchical Reinforcement Learning with Dynamic Recurrent Mechanism (HELAR). Their study attained a breakthrough at the algorithmic level. It invokes learners’ current knowledge reserves through context awareness and explores potential preferences based on dynamic baselines, effectively balancing exploitation and exploration in the recommendation process. Experimental results demonstrate that HELAR outperforms state-of-the-art course recommendation methods (Lin et al., 2022). In order to further tackle the challenges of scarce labeled data and the multiple preferences profiling, subsequently, Lin YG et al. advanced a joint learning framework-Reinforced Profile for Personalized Recommendation (RPPR). By means of a multivariate perceptron attention mechanism, this framework enables adaptive adjustment strategies with limited information, significantly enhancing the multidimensionality of profiling construction and the accuracy of recommendations from a data mining-oriented perspective (Lin et al., 2023).

##### 3.3.2.2 Computational feature analysis and SRL modeling

This field concentrates on applying machine learning and natural language processing technologies to conduct quantitative assessments of learners’ cognitive characteristics, personality traits, and metacognitive behaviors, for the purpose of elevating the interpretability of systems and the targeting of instructional interventions. In the aspect of computational feature analysis, scholars are committed to extracting learner features from unstructured data. Shahbazi et al. established an intelligent agent-based recommendation system by integrating machine learning and NLP technologies, thus realizing the automatic mining of learner styles and individual characteristics (Shahbazi and Byun, 2022). Takami et al. introduced a psychological perspective. Founded on Big Five personality theory, they identified typical profiles (such as diligent type and fear type) through cluster analysis, and built an interpretable personalized recommendation system (Takami et al., 2023). Additionally, Kaur et al. combined core parameters including educational background and knowledge level, and optimized the dynamic adaptability of profiles through a user feedback loop. In terms of SRL, research focuses on revealing the heterogeneity of learner self-regulatory capabilities (Kaur et al., 2022). Tang et al. adopted the two-parameter item response theory fused with cluster analysis to precisely identify learner profiles at different SRL development stages, hence providing a psychometric basis for the design of differentiated Scaffolding (Tang and Bao, 2024). Wu et al. further extended the scenario to Project-Based Learning (PBL): they uncovered the dynamic evolutionary trajectories of SRL capabilities among learners with different profiles using Latent Profile Analysis (LPA), and emphasized the necessity of granular support under specific instructional scenarios (Wu, 2024).

##### 3.3.2.3 Learning analytics-driven resource adaptation, feature profiling, and precision intervention

Learning analytics research revolves around two core directions: precise resource adaptation, and from data technology breakthroughs to educational practice implementation, providing solid support for personalized education. In the research on precise resource adaptation within the MOOCs ecosystem, scholars built targeted solutions to attain efficient resource matching. Confronted by the choice dilemma caused by massive course resources, Campos et al. proposed Fragmented Recommendation for MOOCs Ecosystems (FReME), which harness learning analytics technology to accurately identify learners’ knowledge gaps, generates fragmental content recommendations by integrating multi-platform resources, and optimizes knowledge acquisition paths (Campos et al., 2022). For the extreme scenario of pure cold start without prior interaction data, Butmeh H developed a hybrid recommendation system fusing knowledge attributes and collaborative filtering mechanisms to break the traditional reliance of collaborative filtering on historical ratings. The system achieves precise knowledge mapping between user characteristics and learning material attributes in a two-dimensional space based on Rogers-Tanimoto and Jaccard similarity metrics. The experimental results of 82% recommendation satisfaction and 90% profile matching accuracy fully confirm the significant efficacy of attribute-based knowledge modeling in solving cold start problems (Butmeh and Abu-Issa, 2024).

To overcome the limitation of traditional Latent Analysis (LA) research that excessively relies on explicit outcome data, relevant studies have utilized Latent Class Analysis (LCA) and large-scale trajectory data to construct normative models capable of establishing mapping relationships between objective behaviors and subjective psychological states (Seo et al., 2025; Barthakur et al., 2021). Meanwhile, in response to the common challenges of non-interpretability in LA models, eXplainable Artificial Intelligence (XAI) and anomaly detection technologies have been introduced, which significantly improve the ability to identify and attribute atypical learning patterns and abnormal trends (Tiukhova et al., 2024). In the dimension of learner heterogeneity modeling, research took clustering technology to deepen the quantitative characterization of individual differences in LA. Particularly for the key indicator of self-regulated learning (SRL), the K-means algorithm has been applied to classify exact typical profiles such as “comprehensive type” and “disillusioned type,” this reflects the underlying correlations between learners’ regulatory strategies and academic outcomes across different cultural backgrounds (Tang and Bao, 2024). Ultimately, in the dimension of transforming analytical results into teaching practices, research attention returned to establishing a complete closed loop from data analysis to instructional intervention. Whether it is the integrated system design based on Bloom’s Taxonomy of Educational Objectives, covering goal setting, profile modeling, and intervention implementation (Li et al., 2024), or the use of ontological semantic frameworks to solve the data scarcity problem in cold start scenarios for personalized recommendations (Joy et al., 2021), both have realized the conversion of technical insights from learning analytics into scalable, implementable, and precise teaching support.

The second category is intelligent technology research, in which #3 data mining includes technology, self-regulated learning, machine learning, collaborative filtering, etc.; and #5 cluster analytics contains keywords such as Learning Analytics, Learner Analytics; #7 artificial intelligence covers deep learning, dynamic system, learning system, adaptive educational systems, and so on. Data mining refers to the process of extracting implicit, unknown, and potentially valuable information or patterns from large amounts of data stored in databases, data warehouses, or other information repositories. Clustering is a common data analysis tool, the purpose of which is to divide a large collection of data points into a number of classes, so that the data in each class are maximally similar to each other and the data in different classes are maximally different. In the process of multimedia information retrieval and data mining, clustering processing has important theoretical and practical significance for establishing efficient database indexing and realizing fast and accurate information retrieval. Cluster analysis is used to group students based on similar characteristics in order to identify the needs and behavioral patterns of specific groups. This method allows educators to better engage in personalized teaching, resource allocation, and intervention measures, thereby enhancing educational effects. Based on the above technical foundation, related research intensively integrates theories and technologies including machine learning, self-regulating learning, and adaptive systems, which intends to retrieve potential patterns from massive educational data to achieve precise profiling of learners’ behaviors and intelligent upgrades in teaching intervention. Current research in this field can be summarized into two core academic areas according to the differences in research focus and theoretical framework: Educational Data Mining (EDM) and, as well as Artificial Intelligence applications in EDucation (AIED) and ecological reconstruction.

##### 3.3.2.4 Educational data mining: academic prediction and quality monitoring

Research primarily endeavors to deal with the core educational issue of academic performance prediction using EDM technology, and to realize dynamic monitoring of educational quality through process mining. Researchers have adopted machine learning algorithms such as K-Nearest Neighbors (KNN), J48, and Bayesian networks as the main driving force, aiming to construct a comprehensive prediction model to uncover the underlying correlations between learning processes and outcomes.

Regarding academic prediction, research seeks to early identification of academic risks through the integration of multi-dimensional data. For instance, Yagci Mustafa took undergraduate students as the research subjects and created a new machine learning model using quantitative data like mid-term grades. Eventually, it achieved an accuracy rate of 70–75% in predicting final grades, which established an effective academic prediction method and offered strong support for decision-making optimization in higher education (Yağcı, 2022). Nayak et al. (2023) further strived to impeove the accuracy of student outcome prediction and the efficiency of educational interventions in online courses within the framework of Outcome-Based Education (OBE). Their study compared algorithms involving Decision Tree (J48), Naive Bayes (NB), Random Forest (RF), and Multi-Layer Perceptron (MLP), and combined information gain for feature selection. Empirical results indicate that the optimized MLP model achieves an accuracy of 97.08%, while the Random Forest model incorporating student behavioral features even attains 100% prediction accuracy—providing robust evidence that classroom behavioral features are key variables determining academic outcomes (Nayak et al., 2023). The application of EDM has been further extended to anomaly detection in curriculum evaluation processes. Vaidya Anagh and Sharma Sarika et al. proposed an operable analytical model using learning analytics and educational data mining technologies, attempting to detect anomalies in curriculum evaluation (e.g., persistently underperforming courses) (Vaidya and Sharma, 2024). This model successfully recognized data points deviating from normal behavioral patterns and served as a direct empirical support for ensuring the accuracy of educational outcomes and implementing targeted corrective measures.

##### 3.3.2.5 Artificial Intelligence applications in education: human-machine collaboration and ecological reconstruction

Under the research perspective of Profile Technology Applied in Education (PTAE), research on artificial intelligence education in the past 3 years has shown a profound evolution: from single behavior modeling to the deepening of multi-modal interaction and emotional profiling; from resource recommendation to metacognitive self-regulation support; and from technology application to the reconstruction of the human-machine collaborative education ecosystem. More specifically, the research deeply integrated algorithms like random forests, Long Short-Term Memory networks (LSTM), and natural language processing. It not only established a dynamic portrait covering multiple dimensions including online interaction, language ability, and psychological attitude (Huang X. et al., 2023; Wang and Song, 2024), recently, but also further expanded to the emotional and autonomous profiling based on the extended SOR theory and the dynamic interaction profiling in the generative artificial intelligence environment and thus achieving a deep portrait of learners’ heterogeneity (Liu et al., 2025; Güner and Er, 2025). Building on this foundation, subsequent study commits to tackling motivation stimulation and strategy regulation difficulties in individualized learning. Beyond boosting participation and trust through explainable recommendation systems (Huang A. Y. et al., 2023; Takami et al., 2023), particularly amid the challenges posed by generative artificial intelligence, the research not only theoretically advocates for the establishment of a human-machine collaborative regulation network to protect learners’ subjective initiative (Lodge et al., 2023) but also empirically validates the critical efficacy of metacognitive support in reducing cognitive load, improving technology acceptance, and preventing the decline of self-regulation capabilities (Xu et al., 2025). Furthermore, the research scope extends to the systemic adaptation of educational ecosystems: it constructs a teacher portrait encompassing cognitive, emotional, and skill dimensions based on the micro-ecosystem theory (Hu et al., 2024), and integrates the Unified Theory of Acceptance and Use of Technology 2 (UTAUT2) and General Extended Technology Acceptance Model for E-Learning (GETAMEL) models to disclose the psychological driving mechanisms underlying Science, Technology, Engineering, Mathematics (STEM) teachers’ adoption of AI tools (Ateş and Gündüzalp, 2025). It explicitly calls for policy-coordinated efforts to promote the ethical governance and effective application of AI in education (Xu et al., 2025), which strive to establish a novel data-driven, human-machine collaborative regulatory, and ecologically adaptive digital education system.

The third category is diverse learning scenarios research that is primarily about #4 blended learning and #8 distance learning, which contain key words: “higher education,” “online learning,” “performance,” etc. These key words revolve around three core directions-ecological governance, model integration and process optimization, aiming to systematically reveal the complete evolution rules of the two teaching modes from macro institutionalized strategic deployment to micro teaching practice implementation.

At the level of macro institutional governance and the psychological motivations of the subjects, the research core lies in solving the institutionalized problem of the deep fusion of blended learning from a marginal auxiliary role to a key educational system. Hernan Galvis Alvaro, from the perspective of systemic change in higher education institutions, build a multi-dimensional decision-making framework covering strategic planning and tactical execution through critical analysis, his work acted as path guidance for the deep integration of blended learning and the existing teaching system (Galvis, 2018). To ensure the long-term operation of this hybrid ecosystem, Wu Jen-Her and Ibrahim Mohammed Mansur et al., respectively, approached from the dual subjects of student-and-teacher, they confirmed that computer self-efficacy, interactive atmosphere and internal-and-external motivations are the core factors actuating the continuous application of Blended E-Learning System (BELS) (Wu et al., 2010; Ibrahim and Nat, 2019). At the level of meso-micro teaching practice and support mechanisms, research responded to the typical challenges of hybrid and remote environments based on empirical evidence. On account of the fusion of MOOCs and traditional physical classrooms, Antonia et al. analyzed and clarified the advantages and implementation obstacles of blended teaching in actual implementation through qualitative research methods (Bralić and Divjak, 2018). Karalar Halit et al. gave close attention to the practical needs of the transition to remote learning, they concentrated on the prediction of students’ academic failure risks in a blended learning environment, incorporated synchronous and asynchronous learning activities and multi-dimensional student characteristics, and constructed an integrated model for academic risk prediction, providing empirical support for precise intervention in a remote environment (Karalar et al., 2021).

In response to the high demands on learner autonomy posed by the blended learning model, Lee, Hsin Yu proposed the Intelligent scaffold strategy based on guided logic guidance: ChatGPT-assisted Learning Aid (GCLA), which optimizes the interaction logic between students and ChatGPT. It proved that the mechanism of independent inquiry first and intervention later can significantly improve students’ SRL ability and higher-order thinking level in blended courses, and gain advanced deep learning in a technology-enhanced environment (Lee et al., 2024).

#### 3.3.3 Research trend analysis

**FIGURE 6:**
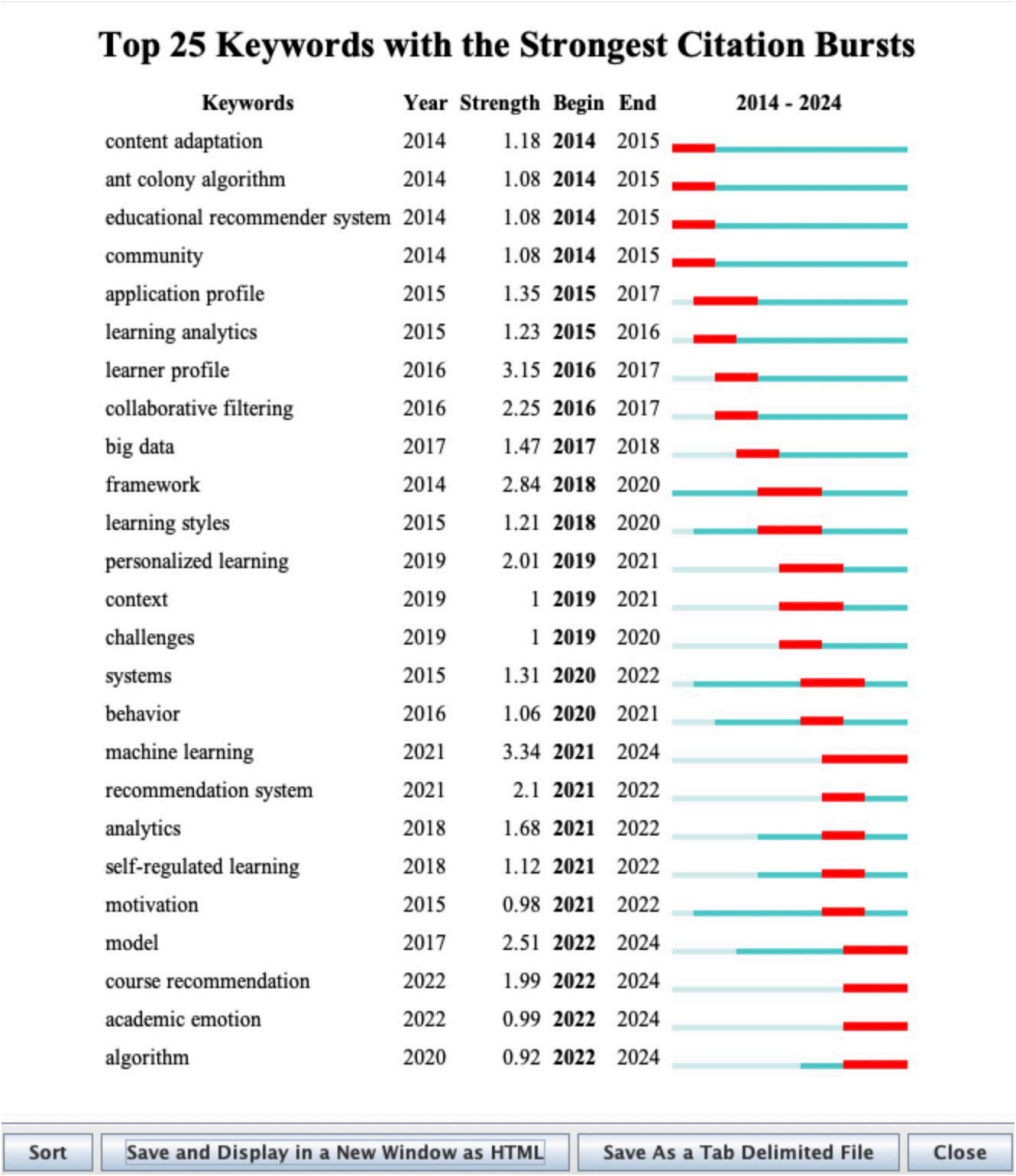
Keyword emergence intensity and time span graph.

To explore PTAE research evolution trends, its core academic value lies in clarifying the intrinsic logic of the theoretical system in this field, as well as the evolutionary context and development direction of its technological forms. The emergence degree of keywords can be used to analyze research areas with significant influence over a period of time, and to display the span between the first and last appearance of the keyword, which helps researchers better discuss the development trend of the field (Wang and Sun, 2020). The keyword time zone map vividly mirrors the evolution of research hotspots over the timeline, facilitating an accurate grasp of the development dynamics of research hotspots from a macro perspective. According to the keyword emergence graph (Figure 6) and the keyword time zone graph (Table 7) drawn by Citespace software, this study finds that the application of Profile Technology in education exhibits a clear three-stage evolution feature.

**TABLE 7:** Research frontier emerging keywords, burst years and cluster attribution table.

Keyword	Burst year	Cluster
Academic emotion	2022–2024	#0 Learning analytics
Course recommendation	2022–2024	#1 Recommender system
Model	2022–2024	#7 Artificial intelligence
Deep learning	2021–2024	#3 Data mining

**FIGURE 7:**
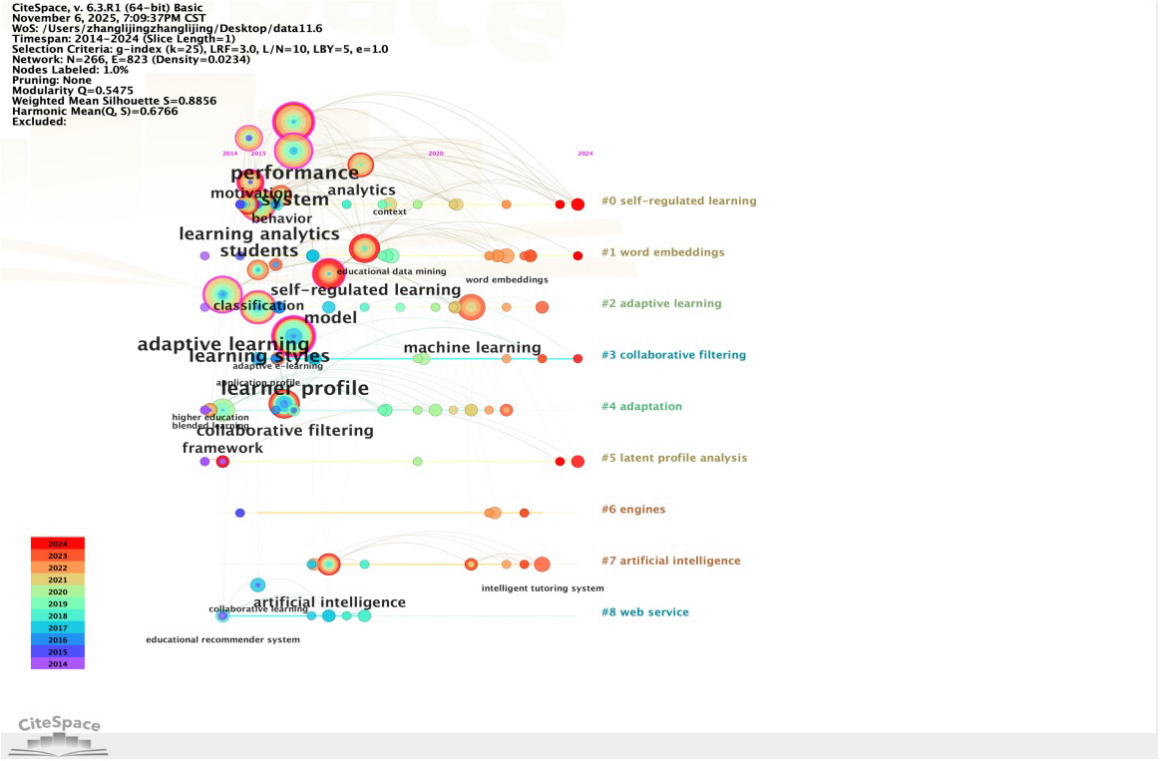
Keyword temporal distribution and evolutionary context map.

A comprehensive analysis of the two graphs (Figures 6, 7) reveals that the evolutionary path of research can be roughly divided into 3 phases. The first stage is from 2014 to 2016, which is the beginning part including the top three emerging terms: “learner profile,” “collaborative filtering,” “application profile.” At this stage, research mainly focuses on infrastructure construction and multi-dimensional learner feature profiling in online learning, with a core emphasis on personalized support and adaptive system design in MOOCs.

Researchers are committed to building multi-dimensional learner profiles covering learning styles, psychological attitudes and emotional characteristics, and are actively exploring the dynamic profile update mechanism (Premlatha et al., 2016). Meanwhile, basic algorithms like collaborative filtering have become the main technical means for achieving individualized resource recommendation and optimization (Bourkoukou and El Bachari, 2016). This indicates that portrait modeling and data analysis technologies have begun to penetrate and be applied in diverse educational scenarios, forming an early design concept that takes into account the psychological characteristics of learners and the precise matching ability of resources, laying the foundation for the subsequent intelligent development of Human-Machine Collaboration.

The second stage, spanning from 2017 to 2021, marks a period of rapid development. With a total of 10 emergent words containing “big data,” “personalized learning,” “machine learning,” etc. Among them “machine learning” has the strongest prominence and occupies a core hub position in co-occurrence network, indicating that as a core engine of Artificial Intelligence, machine learning is promoting research focus to shift from the early basic framework construction to accurate recognition and prediction relying on computational intelligence algorithms. The academic value of this phase resides in the establishment of a data-driven precise intervention mechanism. The research scope zeroes in on the fields of Educational Data Mining (EDM) and Learning Analytics (LA). Scholars extensively adopted machine learning and deep learning techniques to conduct in-depth mining of multi-source data such as Learning Management System (LMS) interaction logs and demographic data, striving to make high-precision academic performance prediction models. The research objective has switched from mere system architecture design to more practical early identification and accurate warning of academic risks (e.g., academic failure or dropout) (Karalar et al., 2021), while simultaneously exploring algorithmic optimization solutions for the cold start problem (Joy et al., 2021). This transformation not only provides a solid evidence-based foundation for scientific educational interventions but also achieves the in-depth adaptation of Profile Technology to personalized and scenario-based educational practices.

The third phase, from 2022 to 2024, is a period of steady research improvement with emergent words “model,” “course recommendation,” “academic emotion,” and “algorithm.” The core academic characteristic of this period lies in embodying the return of technology to a people-centered orientation. From the perspective of mapping features, research themes have gained qualitative breakthroughs in multimodal data integration, affective computing, and intelligent technology closed-loop systems. The research emphasis of this period moved toward the application and reinforcement learning (e.g., actor-critic hierarchical reinforcement learning models (Liang et al., 2023), knowledge graph, deep learning and other advanced algorithms), constructing learner-centered profound profiles. It not only employs latent class analysis and process mining to analyze learners’ self-regulated learning (SRL) strategies and technical attitudes toward generative AI (e.g., ChatGPT) (Wu, 2024; Ateş and Gündüzalp, 2025) but also incorporates academic emotions into the core analytical dimensions (Liu et al., 2025). Building on this foundation, the research endeavors to develop precise recommendation systems capable of solving information overload and noisy data (Campos et al., 2022), and has improved the data support chain from early risk warning to teaching intervention—indicating a complete technical closed loop and educational application logic that ranges from in-depth understanding of learner heterogeneity to comprehensive empowerment of personalized learning processes.

## 4 Conclusion and discussion

Based on a bibliometric and knowledge graph analysis of data from the Web of Science Core Collection, this study systematically reveals the PTAE evolutionary context and cutting-edge landscape between 2014 and 2024. Research findings indicate that the core research paradigm in this field is undergoing a profound ontological and methodological transformation—shifting fundamentally from early static feature description to a dynamic precise intervention mechanism underpinned by a panoramic learning analytics framework. Such change constitutes the underlying logic of modern adaptive learning systems: through the in-depth integration of multimodal intelligent algorithms like Random Forest, Long Short-Term Memory (LSTM) networks, and Natural Language Processing (NLP), real-time perception and refined characterization of learners’ online interaction behaviors, cognitive states, and emotional experiences have been achieved. However, with the improvement of profiling accuracy and the expansion of application scenarios, algorithmic fairness and data privacy protection have emerged as unavoidable ethical challenges. The inherent black-box nature of deep learning algorithms may give rise to implicit biases and undermine educational equity. Therefore, future technological breakthroughs must deeply integrate eXplainable Artificial Intelligence (XAI) technologies to improve system transparency and user trust. Against this backdrop, this study emphasizes the urgency of collaborative governance between educational policies and AI ethics and recommends that policymakers establish clear data governance norms. Particularly in the context of the widespread application of generative AI, efforts should be made to promote the development of an ethical application mechanism for Human-Machine Collaboration. Meanwhile, educators should leverage metacognitive scaffolding mechanisms to improve the accuracy of profiling and safeguard learners’ agency in the learning process through ethical regulation. This will boost the focus of teaching to shift from mere knowledge indoctrination back to the fundamental cultivation of learners’ self-regulation capabilities, and ultimately realize the return of technological logic to the essence of education and create an ecologically adaptive new environment for intelligent education.

### 4.1 Limitations

This study only selected the Web of Science Core Collection as a single data source, with a main focus on journal articles. And this strategy inevitably entails database coverage bias, failing to incorporate a wide range of potential educational technology literature from databases such as Scopus, ERIC, and Google Scholar, while also omitting important literary resources including dissertations and monographs. Furthermore, the restriction of searches to English has resulted in a significant English-language literature preference, overlooking the important contributions of non-English-speaking regions in localized applications. Therefore, subsequent research shall attempt to integrate multi-source databases for convergent validation and incorporate Chinese, English, and other language literatures to construct a more globally oriented and inclusive knowledge graph. Although relying solely on CiteSpace software for mapping offers advantages in emergent term detection, it is constrained by its underlying algorithmic limitations, potentially leading to tool-specific limitation in network form presentation that fail to fully reflect the complex knowledge topology structure. Meanwhile, the naming of keyword clusters and the division of evolutionary phases rely on researchers’ prior knowledge and academic judgment, and the subjectivity of such interpretations may result in deviation in understanding implicit knowledge connections. It is recommended that future research combine the visualization layout advantages of VOSviewer, the statistical depth of the Bibliometrix R package, and introduce qualitative methods such as content analysis or systematic reviews for mixing research. This approach will reduce the subjective bias of manual interpretations, thereby more accurately capturing PTAE mechanisms. From an international perspective, although Profile Technology presents a broad application blueprint globally, its further development still faces multiple profound challenges including but not limited to data ethics, algorithmic fairness, and lagging global collaboration. Specifically, the collection of sensitive data involving learners’ emotions, behaviors, and academic performance touches the boundary between privacy protection and the effectiveness of personalized services. Additionally, the opacity of algorithmic operations may give rise to implicit biases, thereby hindering learners from different cultural and economic backgrounds from equitable access to educational resources and exacerbating structural inequities. Furthermore, the uneven global popularization of technology and geopolitical-cultural differences indicate an urgent need to strengthen current interdisciplinary and cross-regional collaboration networks. In light of this, future research shall not be limited merely to the parameter optimization of technical models but shall extend toward an integrated research framework encompassing technological innovation, ethical governance, and social equity. Thus, to propel the sound and sustainable development of Profile Technology within the global educational ecosystem: establishing a governance mechanism balancing data security and service effectiveness to explore inclusive technological solutions that advance global educational equity is essential; moreover, deepening international interdisciplinary collaboration to collectively address the challenges posed by the digital divide and cultural differences is also necessary.
